# Frailty prevalence according to the Survey of Health, Ageing and Retirement in Europe-Frailty Instrument (SHARE-FI) definition, and its variables associated, in patients with symptomatic knee osteoarthritis: findings from a cross-sectional study

**DOI:** 10.1007/s40520-020-01667-0

**Published:** 2020-07-30

**Authors:** Fausto Salaffi, Marco Di Carlo, Marina Carotti, Sonia Farah, Andrea Giovagnoni

**Affiliations:** 1 Rheumatology Clinic, Università Politecnica Delle Marche, Ospedale “Carlo Urbani”, Via Aldo Moro, 25, Jesi, 60035 Ancona, Italy; 2 Radiology Department, Università Politecnica Delle Marche, Ospedali Riuniti, Ancona, Italy

**Keywords:** Symptomatic knee osteoarthritis, Frailty, Pain, Comorbidity, Polypharmacy

## Abstract

**Background:**

Frailty is a frequent condition in patients with knee osteoarthritis (KOA). However, there are different constructs on how to define it. Survey of Health, Ageing and Retirement in Europe-Frailty Instrument (SHARE-FI) is one of them.

**Aim:**

To assess the prevalence of frailty, according to the SHARE-FI definition in patients with symptomatic KOA, and to establish its associated factors.

**Methods:**

Symptomatic KOA patients were evaluated for pain symptoms, quality of life, comorbidities, ongoing drug therapy, and radiological damage. Patients were categorised according to the SHARE-FI definition into frail, pre-frail, and non-frail, and compared to a group of healthy controls associated by age and gender.

**Results:**

170 symptomatic KOA patients (76.5% female, mean age 70.1 years) and 186 healthy controls were included. According to SHARE-FI criteria, 35 patients (20.6%) were categorised frail, 50 (29.4%) pre-frail, and 85 (50%) non-frail. The prevalence of frail or pre-frail subjects was statistically significantly higher in patients with symptomatic KOA. Stratifying the patients according to the frailty categories, frail subjects showed significantly higher mean values of pain. The results from logistic regression analysis revealed that polypharmacy (*p* = 0.003), pain (*p* = 0.016) and comorbidities (*p* = 0.035) were the variables independently associated with frailty in symptomatic KOA.

**Discussion:**

Frailty or pre-frailty, defined by SHARE-FI, is common in symptomatic KOA. The main factors associated with frailty were polypharmacy, pain and comorbidity burden.

**Conclusions:**

SHARE-FI can represent an useful tool to define frailty in symptomatic KOA.

## Introduction

Knee osteoarthritis (KOA) is the most prevalent chronic joint disease in the world, and one of the most common sources of pain [1]. Especially in the elderly, KOA is a cause of reduced function, generally concerning mobility and is an important cause of frailty and pre-frailty [2].

In recent years frailty, defined as “a biologic syndrome of decreased reserve and resistance to stressors, resulting from cumulative declines across multiple physiologic systems, and causing vulnerability to adverse outcomes” [3], has emerged as a significant area of research in rheumatology [4–6].

KOA is associated with accelerated biological aging and, as a result, geriatric syndromes like frailty are more likely to present irrespective of chronological age. Data from the European Project on OSteoArthritis (EPOSA) demonstrated that frailty and pre-frailty were, respectively, present in the 10.2% and in the 51.0% of the KOA patients. The odd ratio for developing frailty was 2.96 in KOA patients [7]. Pain is a major contributor in developing frailty in KOA patients. A recent study demonstrated that after adjusting for potential confounders (age, gender, anthropometric and demographic data, comorbidities), people with KOA and pain were significantly more likely to have frailty compared with those with KOA without pain [8].

The association between frailty and polypharmacy is also close in the elderly patient [9, 10]. While there are reports of an association between frailty and polypharmacy, causality has not been demonstrated [11]. A large Australian study has revealed that polypharmacy increases the risk of frailty by more than two times [10]. A better understanding of the relationship between KOA, polypharmacy, and frailty is a key challenge from both a clinical and public health perspective. Identifying risk factors for the development of frailty, in a prevailing condition such as KOA, could guide preventive actions in the most susceptible subjects.

On the other hand, there is no consensus on the frailty definition. To date, there are no studies evaluating the prevalence of frailty in symptomatic KOA according to the Survey of Health, Ageing and Retirement in Europe-Frailty Instrument (SHARE-FI) definition [12].

Based on these considerations, the aims of this study were to determine the prevalence of frailty from the Research on Osteoarthritis Against Frailty (ROAF) study, and to determine the factors associated with frailty in elderly patients with symptomatic KOA, using the SHARE-FI.

## Methods

### Subjects

The study population was composed of subjects enrolled in the ROAF study, a study cohort of OA adult patients whose particular focus was to assess the prevalence of frailty and to establish its potential associated independent and combined factors. The patients of the ROAF study enrolled at the Rheumatology Clinic of the Università Politecnica delle Marche from 2018. Patients with symptomatic KOA were consecutively included, diagnosed based on clinical history and objective examination. Pain was the entry criterion, evaluated by the Italian version of the Western Ontario and McMaster Universities OA Index (WOMAC) pain subscale score [13], and in addition three of the following criteria: age ≥ 50 years, morning stiffness < 30 min evaluated by the WOMAC stiffness subscale (score from ‘mild’ to extreme’), crepitus on active motion in at least one knee, bony tenderness in at least one knee, bony enlargement in at least one knee, and no palpable synovial fluid collections or warmth in both knees [14]. All participants were independent in their activities of daily life. Patients on drug treatment for pain with analgesics or non-steroid anti-inflammatory drugs had to be at a stable dosage for at least 2 weeks.

Exclusion criteria were the presence of a coexisting inflammatory joint disease, of medical comorbidities that would render the patient unable to participate fully in study procedures (e.g., terminal conditions such as end-stage renal disease, heart failure or malignancy), alcohol abuse, psychiatric disorder, or previous or planned knee arthroplasty.

Data for the healthy control group were collected from a previous cross-sectional population-based study, called MArche Pain Prevalence INvestigation Group (MAPPING). This study has been described in detail elsewhere [1, 15]. The data collected from 186 healthy controls was used in this study. This sample reflects the age/gender related stratification/distribution of the KOA population under study.

### Frailty definition

The definition of frailty used refers to the variables of the SHARE-FI study [12]. The SHARE-FI instrument was created as per the standard procedure [16]. In SHARE-FI, the cut-offs of the frailty categories (i.e., non-frail, pre-frail and frail) are based on latent variable modelling. The variables previously selected by Santos-Eggimann and coworkers were included [17], and were the following. “Exhaustion” was identified with the question: "In the last month, have you had too little energy to do the things you wanted to do?". A positive answer coded as 1, a negative answer as 0. “Weight loss” was identified by reporting a "Diminution in desire for food" in response to the question: "What has your appetite been like?" or, in the case of a non-specific or uncodeable response to this question, by responding "Less" to the question: "So, have you been eating more or less than usual?". The presence of the criterion was coded as 1, its absence as 0. “Slowness” was defined as a positive answer to either of the following two items: "Because of a health problem, do you have difficulty [expected to last more than 3 months] walking 100 m?" or "… climbing one flight of stairs without resting?". One or two positive answers were scored 1, and two negative answers were scored 0. “Low activity” was assessed by the question: "How often do you engage in activities that require a low or moderate level of energy such as gardening, cleaning the car, or doing a walk?". This variable was kept ordinal: 1 = "More than once a week"; 2 = "Once a week"; 3 = One to three times a month" and 4 = "Hardly ever or never". "Weakness" was the only criterion that required instrumental measurement, for which a five-sensor electric dynamometer (FSR-402) was used, connected to a microprocessor Arduino Mega 2560, already employed by our group both in the context of rheumatoid arthritis and fibromyalgia [18]. This variable was kept as continuous, and the other variables were calculated as indicated in the validation work [12].

The parameters above mentioned allowed the calculation of the SHARE-FI. Its translated calculators (one for each sex) are freely accessible on https://sites.google.com/a/tcd.ie/share-frailtyinstrument-calculators/. When data are entered into the calculator, the tool provides a continuous frailty score and enables automatic classification into phenotypic frailty categories.

SHARE-FI has proven to be a tool with good construct and predictive validity [12]. It offers a quick and reliable way to assess and monitor frailty in community dwelling individuals over the age of 50, can help to prioritise subjects access to resources, and can serve as an instrument for audit and research [19].

### Demographic, clinical and radiographic variables

Clinical and demographic variables were collected during outpatient visits.

The educational level was considered counting the school years from the first year of primary school.

Body mass index (BMI) was categorised accordingly as normal (18.5 to < 25 kg/m^2^), overweight (25 to < 30 kg/m^2^), and obesity (≥ 30 kg/m^2^).

Polypharmacy has been defined by the presence of a therapy with between five and nine drugs; more than 10 drugs define excessive polypharmacy [9, 10].

The comorbidity burden has been defined with modified Rheumatic Disease Comorbidity Index (mRDCI), calculated with the formula: 1* lung disease and [2* (myocardial infarction, other cardiovascular diseases, or stroke) or 1* hypertension] and 1* (ulcer or other gastrointestinal diseases) and 2* kidney disease and 1* BMI > 30 or 2* if BMI is > 35, and 1 for each of diabetes, fracture, depression and cancer. The index has proved its validity and has already been used in various conditions of rheumatological interest [20].

Pain due to KOA was evaluated with the Western Ontario and McMaster Universities Osteoarthritis Index (WOMAC) and its subscales [13, 21].

The Italian version of the 36-item Short Form Health Survey (SF-36) was used as a generic health-related quality of life (HRQoL) scale, computing the two psychometrically based summary measures Physical Component Summary Scale Score (PCS) and Mental Component Summary Scale Score (MCS) [22].

X-rays of the knees were evaluated in the antero-posterior, weight-bearing, semiflexed views. Recent (within one year) images were scored by a musculoskeletal radiologist (MC) according to the Kellgren and Lawrence (K/L) grading system [23].

### Statistical analysis

Data were stored in a Microsoft Excel database and have been processed with MedCalc 19.0.6 (statistical software packages for Windows XP). Parametric techniques may be applicable for certain ordinal level data; however, our data were generally normally distributed. The Kolmogorov–Smirnov test was used to determine the normal distribution. The data were generally normally distributed and, however, presented for the sake of exhaustiveness as means and standard deviations (SDs) and as median and interquartile ranges (IQR). In accordance with the SHARE-FI calculation, patients have been categorised into frail, pre-frail or non-frail.

In the symptomatic KOA patients group, the frailty phenotype comparison (dependent variable) was tested with the chi-square test or Fisher’s exact test for comparison with categorical variables. The non-parametric Spearman’s rank correlation coefficient was used to assess the relationships between clinical, functional and radiological measures and SHARE-FI scores. Differences in participant characteristics between patients and controls and between frailty categories were tested with one-way analysis of variance (ANOVA), or Kruskal–Wallis analysis as appropriate. To assess the relative contribution of the single variable (age, sex, disease duration, level of education, polypharmacy, mRDCI, BMI, SF-36, and radiographic OA severity) on the SHARE-FI score (considered as dependent variable), a multivariate logistic regression analysis in symptomatic KOA patients was performed. Analysis with backward elimination included variables that yielded *p* values of 0.1 or lower in the initial univariate analysis. *p* values < 0.05 were considered statistically significant.

## Results

One-hundred and seventy consecutive symptomatic KOA patients (76.5% females, mean age 70.1 years) were included, 96 (56.5%) complaining symptoms in the right knee and 74 (43.5%) in the left one. The mean duration of KOA symptoms until the diagnosis was 7.5 years (range 1–19 years). The education level was generally low, 43.5% had received only a primary school education, and just 9.4% had received a high school education or more. The majority of the patients (73.5%) were married living family, up to 53.8% were housewives. BMI indicative of overweight were recorded for 59.7% of the patients. The radiological severity of the KOA was predominantly in the second and third degree K/L (respectively, 43.5% and 35.3%). Polypharmacy was very common in our study population, with 53.6% of the subjects receiving 5–9 drugs per day and 13.8% receiving 10 drugs per day or more. Control subjects differed from symptomatic KOA patients for a lower mean comorbid score (mRDCI 3.5 ± 1.9 versus 3.0 ± 2.0; *p* = 0.042), lower daily drug consumption (3.5 ± 1.9 versus 2.5 ± 2.0; *p* = 0.002) and a lower mean BMI value (26.8 ± 3.9 versus 25.7 ± 3.3; *p* = 0.003) (Table 1).

**Table 1:** Summary statistics of demographic, clinical, and radiological features in symptomatic KOA patients (170 patients) and healthy controls (186 individuals)

	Symptomatic KOA	Healthy controls	*p* value
Age (years)			
Mean (SD)	70.1 (7.1)	69.1 (8.2)	0.238
Range	53–81	54–83	
Sex (*n*°, %)			
Male (%)	40 (23.5%)	39 (21.0%)	0.561
Female (%)	130 (76.5%)	147 (79.0%)	
BMI (Kg/m^2^)			
Mean (SD)	26.8 (3.9)	25.7 (3.3)	0.003
Range	17.9–42.8	18.1–44.2	
Educational level (years)			
Mean (SD)	10.5 (6.6)	11.4 (3.8)	0.124
Range	5–16	6–18	
Pain duration (years)			
Mean (SD)	7.5 (7.1)	–	
Range	1–29	–	
Kellgren/Lawrence rating score, *n*° (%)			
Grade 1	19 (11.2%)	–	
Grade 2	74 (43.5%)	–	
Grade 3	60 (35.3%)	–	
Grade 4	17 (10.0%)	–	
Comorbidities (%)			
0–1	(48.6%)	(54.8%)	
2–3	(23.6%)	(19.4%)	
≥ 4	(27.8%)	(25.8%)	
mRDCI, mean (SD)	3.5 (1.9)	3.0 (2.0)	0.042
Number of drugs per day (%)			
0–4 drugs	(32.6%)	(42.5%)	
Polypharmacy (5–9 drugs)	(53.6%)	(48.9%)	
Excessive polypharmacy (≥ 10 drugs)	(13.8%)	(8.6%)	
Number of drugs per day, mean (SD)	5.9 (2.8)	4.9 (2.5)	0.002

*BMI* body mass index, *mRDCI* modified Rheumatic Disease Comorbidity Index

**Fig. 1:**
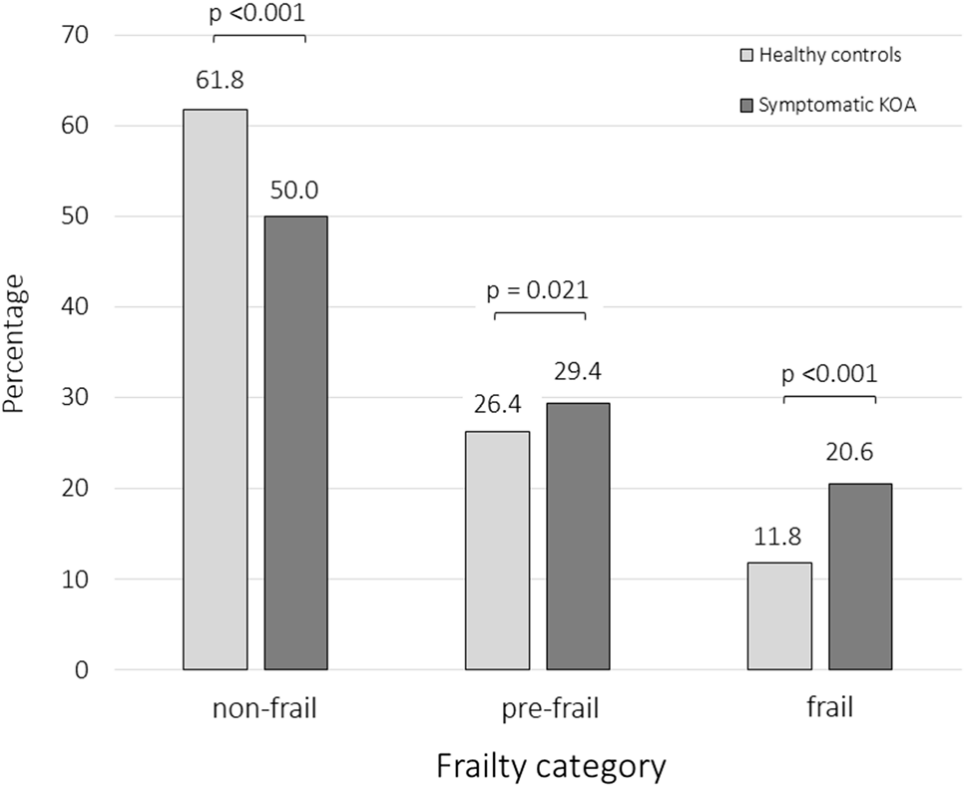
Distribution of frailty categories in symptomatic knee osteoarthritis (KOA) patients (170 subjects) and healthy controls (186 subjects) according to the SHARE-FI definition (comparison with the ANOVA test)

The prevalence of frail or pre-frail subjects was statistically significantly higher in patients with symptomatic KOA. According to SHARE-FI, 35 symptomatic KOA patients (20.6%) were identified as frail, 49 (26.4%) as pre-frail, and 85 (50.0%) as non-frail. In healthy group, 22 subjects (11.8%) were identified as frail, 50 (29.4%) as pre-frail, and 115 (61.8%) as non-frail (chi-squared = 6.77; *p* = 0.033) (Fig. 1).

**Fig. 2:**
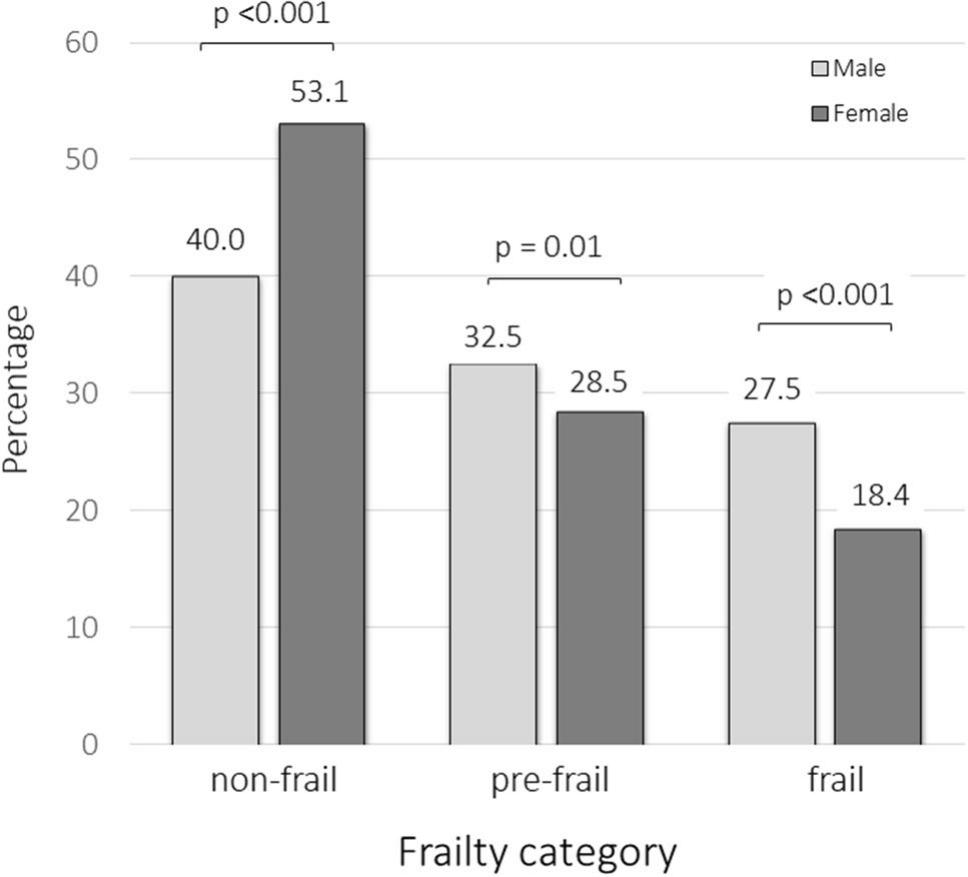
Distribution of frailty categories in symptomatic knee osteoarthritis (KOA) patients according to gender (comparison with the ANOVA test)

By making a gender distinction, male patients were significantly more frail (*p* = 0.036) than female patients (Fig. 2).

**Fig. 3:**
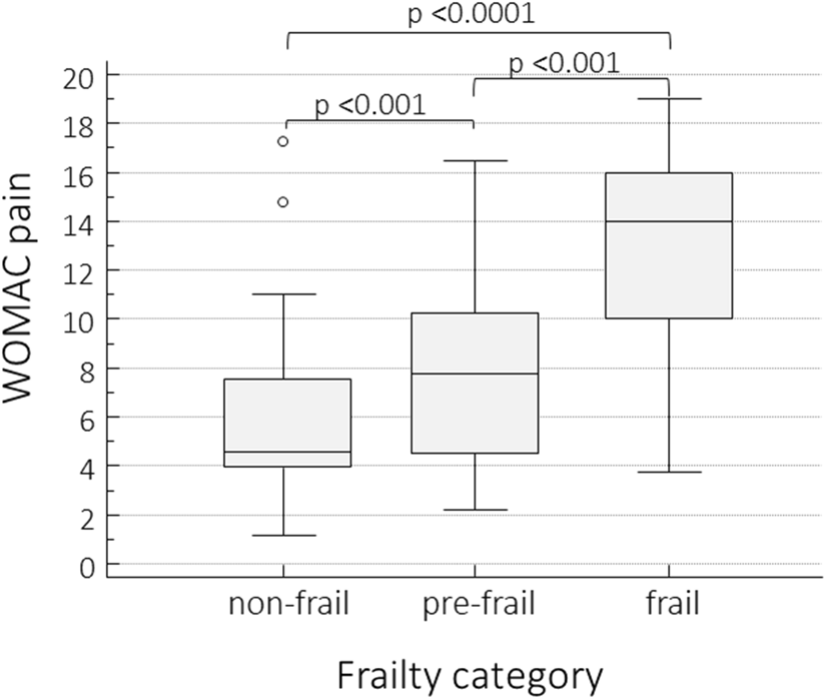
Distribution of the WOMAC pain scores according to the frailty categories in symptomatic KOA patients (comparison with the ANOVA test)

Stratifying symptomatic KOA patients according to the frailty categories, frail patients showed significantly higher mean values of WOMAC Pain (Fig. 3) and SF-36.

The mean number of drugs prescribed increased with increasing frailty in symptomatic KOA patients: 4.1 ± 1.1 in non-frail subjects, 6.0 ± 1.6 in pre-frail subjects, 10.3 ± 1.8 in frail subjects (*F*-ratio = 239.387, *p* < 0.001). Moreover, mRDCI differed significantly between in symptomatic KOA patients classified as frail, pre-frail, and non-frail (*F*-ratio = 73.87, *p* < 0.001) (Table 2).

**Table 2:** Summary statistics of demographic, clinical, and radiological variables according to the three categories of frailty in symptomatic KOA patients

	Frailty category											
	Non-frail				Pre-frail				Frail			
	Mean	Median	SD	25–75 P	Mean	Median	SD	25–75 P	Mean	Median	SD	25–75 P
Age (years)	67.17	68.00	6.94	60.75–71.00	71.24	71.50	6.40	67.00–77.00	75.42	77.00	4.91	73.00–79.00
BMI (Kg/m^2^)	26.56	26.63	3.54	23.49–28.81	26.10	25.72	4.01	23.61–28.30	28.47	27.73	4.58	25.47–28.94
Duration (years)	6.95	5.00	8.36	2.00–8.00	8.40	7.00	6.18	4.00–12.00	7.80	8.00	5.01	4.00–10.75
Education (years)	11.38	9.00	8.39	8.00–13.00	10.62	9.00	3.90	8.00–13.00	8.11	7.00	3.60	5.00–9.00
Kellgren/Lawrence	2.42	2.00	0.72	2.00–3.00	2.56	3.00	0.81	2.00–3.00	2.74	3.00	0.91	2.00–3.00
Polypharmacy	4.08	4.00	1.02	3.75–4.00	6.00	6.00	1.64	5.00–7.00	10.31	11.00	1.84	9.00–11.75
mRDCI	2.49	2.00	1.34	1.00–3.00	3.34	3.00	1.55	2.00–4.00	6.02	6.00	1.54	5.25–7.00
SF-36 MCS	62.13	70.83	22.69	40.00–82.22	54.73	47.00	17.78	41.11–69.88	30.39	29.00	7.53	24.17–36.91
SF-36 PCS	56.07	61.50	20.68	39.00–74.00	50.55	51.00	12.88	46.25–61.25	33.20	25.00	20.37	18.25–49.75
SHARE-FI	0.64	0.33	0.40	0.26–1.04	2.92	2.88	0.69	2.45–3.37	7.58	7.65	0.60	6.98–7.98
WOMAC pain	5.68	4.50	2.80	3.93–7.50	8.17	7.75	3.95	4.50–6.55	12.88	14.00	4.88	10.00–16.00

SD standard deviation, *P* percentiles, *BMI* body mass index, *mRDCI* modified Rheumatic Disease Comorbidity Index, *SF-36* 36-item Short Form Health Survey, *MCS* Mental Component Summary scale, *PCS* Physical Component Summary scale, *SHARE-FI* Survey of Health, Ageing and Retirement in Europe-Frailty Instrument, *WOMAC* Western Ontario and McMaster Universities Osteoarthritis Index

The positive correlations of WOMAC pain were significant with SHARE-FI (rho = 0.445, *p* < 0.0001), polypharmacy (rho = 0.481, *p* < 0.0001), and mRDCI (rho = 0.311, *p* < 0.0001). Conversely, WOMAC pain was negatively associated with physical function and psychological distress (SF-36 PCS and SF-36 MCS scales, respectively, rho = − 0.480; *p* = 0.004 and rho = − 0.256; *p* = 0.026) (Table 3).

**Table 3:** Correlation analysis (Spearman rank correlation coefficient) between the variables studied in symptomatic KOA patients

		BMI (kg/m^2^)	Pain duration (years)	Educational level (years)	Kellgren/Lawrence	Polypharmacy	mRDCI	SF36 MCS	SF36 PCS	SHARE-FI	WOMAC pain
Age (years)	rho p	0.187 0.014	0.198 0.009	− 0.170 0.026	0.183 0.017	0.307 < 0.0001	0.254 0.0008	− 0.347 0.002	− 0.434 0.010	0.451 < 0.0001	0.294 0.0001
BMI (kg/m^2^)	rho p		0.205 0.007	− 0.092 0.232	0.177 0.020	0.111 0.151	0.103 0.182	− 0.167 0.153	− 0.201 0.254	0.117 0.130	0.188 0.013
Pain duration (years)	rho p			− 0.053 0.491	0.224 0.003	0.210 0.005	− 0.004 0.955	− 0.224 0.053	− 0.224 0.203	0.132 0.086	0.285 0.0002
Educational level (years)	rho p				0.009 0.906	− 0.158 0.039	− 0.210 0.009	0.059 0.616	0.176 0.320	− 0.199 0.009	− 0.203 0.008
Kellgren/Lawrence	rho p					0.130 0.092	0.054 0.481	− 0.560 < 0.0001	− 0.482 0.003	0.134 0.082	0.190 0.013
Polypharmacy	rho p						0.514 < 0.0001	− 0.386 0.0006	− 0.380 0.026	0.681 < 0.0001	0.481 < 0.0001
mRDCI	rho p							− 0.272 0.018	− 0.450 0.007	0.554 < 0.0001	0.311 < 0.0001
SF36 MCS	rho p								0.742 0.003	− 0.538 < 0.0001	− 0.256 0.026
SF36 PCS	rho p									− 0.405 0.017	− 0.480 0.004
SHARE-FI	rho p										0.445 < 0.0001

*BMI* body mass index, *mRDCI* modified Rheumatic Disease Comorbidity Index, *SF-36* 36-item Short Form Health Survey, *MCS* Mental Component Summary scale, *PCS* Physical Component Summary scale, *SHARE-FI* Survey of Health, Ageing and Retirement in Europe-Frailty Instrument, *WOMAC* Western Ontario and McMaster Universities Osteoarthritis Index

The results from logistic regression analysis revealed that polypharmacy (Wald statistic = 8.740; *p* = 0.003), WOMAC pain (Wald statistic = 5.828; *p* = 0.016) and comorbidities (Wald statistic = 4.445; *p* = 0.035) were independently associated with frailty in symptomatic KOA (Table 4).

**Table 4:** Multinomial logistic regression analysis (coefficients, standard errors and Wald statistic) of the variables associated with frailty in symptomatic KOA patients

Variable	Coefficient	Standard error	Wald	*p*
Age (years)	− 0.094	0.115	0.670	0.413
Gender	2.91	1.89	2.378	0.123
BMI (kg/m^2^)	0.311	0.153	3.598	0.063
Pain duration (years)	0.160	0.125	1.644	0.200
Educational level (years)	0.013	0.158	0.007	0.935
mRDCI	− 1.285	0.611	4.445	0.035
Polypharmacy	− 1.075	0.365	8.740	0.003
Kellgren/Lawrence	0.009	0.690	0.012	0.989
SF-36 MCS	0.023	0.021	1.965	0.169
SF-36 PCS	− 0.071	0.025	0.021	0.907
WOMAC pain	− 0.405	0.168	5.828	0.016
Intercept	17.623	8.994	3.839	0.052

*BMI* body mass index, *mRDCI* modified Rheumatic Disease Comorbidity Index, *SF-36* 36-item Short Form Health Survey, *MCS* Mental Component Summary scale, *PCS* Physical Component Summary scale, *WOMAC* Western Ontario and McMaster Universities Osteoarthritis Index

## Discussion

In this study, we demonstrated an association between symptomatic KOA and frailty. Compared to healthy subjects, frailty and pre-frailty showed a higher prevalence in patients with symptomatic KOA. Frailty seems to be significantly conditioned by polypharmacy and by the presence of comorbidity in patients suffering from symptomatic KOA. For the first time, the definition of frailty covered by SHARE-FI was applied in patients with symptomatic KOA. The SHARE-FI implies a relative ease of use, combining patient-reported evaluations with objective measures such as the assessment of handgrip strength.

From a medical point of view, the term "frail" identifies patients with a reduced ability to effectively compensate for external stressors, and who are consequently at greater risk of negative outcomes, including prolonged hospitalization, institutionalization, worsening disability, and even death [3]. There is an agreement that the biological basis of frailty is multifactorial. Frailty includes dysfunctions of various systems, its risk increases in a non-linear model according to the number of altered systems, and is independent of chronic diseases and chronological age. In literature, different criteria have been validated to identify frail older subjects, which mainly refer to two conceptual models: the physical frailty phenotype proposed by Fried [3]*,* and the cumulative deficit approach proposed by Rockwood [16, 24].

A number of studies have demonstrated a significant relationship between KOA and frailty [25, 26]. Symptomatic KOA predisposes affected individuals to multiple variables included in the definition of frailty, including sarcopenia, fatigue and low activity. Symptomatic KOA, particularly if characterised by significant painful symptoms, is a condition that the clinician must be able to manage to avoid the appearance or worsening of frailty. This study also investigated the presence of collateral variables involved in the increased risk of frailty, emerging polypharmacy and comorbidities as two conditions that are associated to frailty. In our cohort, we found an overall prevalence of frailty in KOA of 20.6%, a higher rate than described by Fried and colleagues (6.9%) [3] and by other European studies [2, 7]. These differences may depend on several factors, the main one seems to be the definition of frailty itself. From large multicentre and multinational studies, it emerges that frailty affects 10.2% of subjects aged 65–85 years, and OA increases the probability of frailty by a factor of 2.96 compared to subjects without OA [7]. Some differences have also been documented on a geographical basis, finding in Mediterranean countries (Italy and Spain) an increased risk of frailty.

The association between KOA-related pain and frailty can be clarified by a variety of interconnected mechanisms, and has already been demonstrated in several cohort studies, and the presence of moderate or severe knee pain increases the likelihood of pre-frailty or frailty [27]. Bilateral knee pain increases this probability, both for pre-frailty and frailty, compared to monolateral symptomatology.

The fact that the more severe the pain and the greater the condition of frailty also emerges from cross-sectional studies [28]. Persistent pain contributes to and accelerates the onset of frailty or development of frailty in elderly people through mechanisms involving reduced movement ability, decreased nutritional intake, and finally the onset of new comorbidities such as depression [29]. Besides the symptomatology due to KOA, a certain importance is also covered by radiological damage. Radiological damage on a symptomatic KOA would seem to confer an additional risk of frailty [2].

The pain symptom, however, when connoted by particular characteristics of pervasiveness, is the major determinant of frailty within KOA [30], and remains such even without considering associated confounding factors such as depression and the use of opioid drugs. Pain also translates into an increased risk of falling with the consequent unfavourable outcomes [31].

Our study confirmed an already known association between polypharmacy and frailty [3]. On average, frail subjects consume more drugs, and this finding is also directly related to comorbidities. Frail subjects, frequently elderly, suffer from multiple chronic diseases, such as cardiovascular diseases and hypertension, obesity, respiratory diseases, and type II diabetes mellitus [32]. Polypharmacy, defined as five prescribed drugs or more, in our case history covered more than two thirds of the sample. This confirms the existence of a gradient between the number of drugs prescribed and the prevalence of frailty. Polypharmacy and excessive polypharmacy (10 drugs or more) are a common condition in subjects over 75 years, reported with a frequency of 34% and 23%, respectively [33]. After adjustment for socio-demographic and health variables, polypharmacy and excessive polypharmacy were associated with frailty with a probability ratio of 1.77 and 4.47, respectively. Chronic KOA pain is also a predisposing factor for polypharmacy, since it increases the consumption of opioid and non-opioid analgesic drugs [34, 35]. Although analgesics can alleviate painful symptoms, their use in older people is characterised by an increased risk of adverse events (falls, fractures, and delirium) due to the pharmacokinetic and pharmacodynamic changes common in old age [35]. Polypharmacy also increases the risk of inappropriate prescription [36], drug interactions [37], and overall adverse events including functional impairment and hospitalisation [38]. To all effects, polypharmacy is to be considered a factor predisposing to frailty [39].

Finally, when considering the limitations of the study, mention should be made of the cross-sectional assessment, which does not provide evidence of causality. Furthermore, the vast majority of the data collected are patient-reported measures affected by possible recall errors. Another limitation of the study is that the potential effect of analgesic/anti-inflammatory treatment was not considered, recruiting patients with symptomatic KOA both in treatment and out of treatment.

## Conclusions

The results of this study suggest that pre-frailty and frailty, measured with SHARE-FI, are common in the symptomatic KOA patients. Pain, together with comorbidities and polypharmacy are the main determinants of frailty in the course of symptomatic KOA. Further studies are needed to identify in detail the pathophysiological mechanisms of these associations to provide effective treatment strategies.

## Availability of data and material

The data are available upon reasonable request to the corresponding author.
